# Acute and Subchronic Toxicity Profile of a Polyherbal Drug Used in Sri Lankan Traditional Medicine

**DOI:** 10.1155/2020/2189189

**Published:** 2020-07-13

**Authors:** Donisha Shani Niharika Keembiya Liyanagamage, Susanthi Jayasinghe, Anoja Priyadarshani Attanayake, Veranja Karunaratne

**Affiliations:** ^1^Department of Chemistry, Faculty of Science, University of Peradeniya, Peradeniya, Sri Lanka; ^2^Postgraduate Institute of Science, University of Peradeniya, Peradeniya, Sri Lanka; ^3^Department of Biochemistry, Faculty of Medicine, University of Ruhuna, Matara, Sri Lanka

## Abstract

A polyherbal drug composed of leaves of *Murraya koenigii* L. Spreng, cloves of *Allium sativum* L., fruits of *Garcinia quaesita* Pierre, and seeds of *Piper nigrum* L. is a popular drug which has been used by indigenous practitioners in Sri Lanka for the treatment of diabetes mellitus and dyslipidemia. The acute toxicity assessment was conducted, following a single oral dose of 0.25–2.0 g/kg in healthy rats, and rats were observed up to 14 days. The hot water extract (1.0 g/kg) and the water : acetone extract (0.5, 1.0, and 1.5 g/kg) were administered to Wistar rats for 28 days in the subchronic study. Hypoglycemic and antihyperglycemic activities (dose response studies) of cold water, hot water, and water : acetone extracts of the polyherbal mixture were evaluated at the doses of 0.5, 1.0, and 1.5 g/kg in healthy and streptozotocin-induced diabetic rats (70 mg/kg, ip), respectively. Acute toxicity study showed that the polyherbal drug did not cause any change in animals throughout the experimental period of 14 days. The administration of the hot water extract and the water : acetone extract of the polyherbal drug for 28 days did not produce changes in the selected biochemical and hematological parameters in Wistar rats (*p* > 0.05). The histological assessment corroborated the biochemical findings with no significant treatment-related changes in the kidney and liver. The treatment of polyherbal drug significantly lowered the serum glucose concentration compared to the diabetic control rats (*p* < 0.05) while it did not lead to a severe reduction of glucose concentration in healthy rats. The hot water and water : acetone extracts of the polyherbal drug showed a statistically significant improvement on total area under the glucose tolerance curve in diabetic rats (*p* < 0.05), reflecting dose-dependent antihyperglycemic effects of the drug. Based on the results, we conclude that the aforementioned antidiabetic polyherbal remedy is free of toxic/adverse effects at the equivalent human therapeutic dose in healthy Wistar rats and would be a safe therapeutic agent for long-term treatments.

## 1. Introduction

Different types of noncommunicable diseases such as diabetes mellitus have been treated with many traditional medicines which are derived from medicinal plants [1]. However, the major hindrance in incorporation of herbal medicine in modern medical practices is lack of scientific and preclinical data proving their efficacy and safety. A polyherbal drug made of leaves of *Murraya koenigii* L. Spreng (Family: Rutaceae), cloves of *Allium sativum* L (Family: Amaryllidaceae), dried fruit rinds of *Garcinia quaesita* Pierre (Family: Clusiaceae), and seeds of *Piper nigrum* L. (Family: Piperaceae) has been prescribed by many indigenous practitioners in Sri Lanka in the treatment of diabetes mellitus and dyslipidemia [2, 3]. In addition, ingredients of the above mixture are well-known antidiabetic herbal therapeutics in traditional medicine that have been extensively used to manage diabetes not only in Sri Lanka but also in many South Asian countries. Recent scientific studies have disclosed their evidences for potential antidiabetic activity of its ingredients: leaves of *M. koenigii*, cloves of *A. sativum*, *P. nigrum*, and their isolated compounds [4–9]. Although there is no scientific data on antidiabetic activity of dried fruit rinds of *G. quaesita*, plants of this genus such as *Garcinia pedunculata, Garcinia indica*, and *Garcinia xanthochymus* have been reported for their significant antidiabetic activities *in vivo* [10–12]. The antidiabetic efficacy of the polyherbal drug has not been scrutinized *in vivo*; however, it needs to be balanced with the safety particularly when the drug is being properly recommended for long-term treatments.

Despite the presence of therapeutically beneficial secondary metabolites, a number of toxic phytochemical constituents are found in plants. Chronic use of these plant-based therapeutic agents may lead to chronic adverse/toxic effects mainly renal and hepatic toxicities in humans [13]. Emerging reports have indicated that the frequent intake of certain plant remedies used in traditional medicine may result in harmful effects mostly at higher doses than the therapeutic dose [14]. Sri Lankan traditional medicine utilizes plethora of endemic and native medicinal plants to formulate vast range of herbal remedies. However, Arseculeratne et al. [15] have revealed that some of the Sri Lankan medicinal plants used in traditional medicine contain pyrrolizidine alkaloids that could exert considerable hepatic and renal toxicities. Even though toxic effects of the herbal medicines are negligible at low doses and occasional treatments, their utilization at high doses and more importantly the chronic usage for an undefined period might be able to develop adverse side/toxic effects. This was observed in the toxicity studies reported by the Fowotade et al. [16] for the *A. sativum* extract where the low doses of *A. sativum* extract (250 mg/kg body weight/day) exerted no deleterious effects on the organs of the Wistar rats while high doses (>400 mg/kg body weight/day) led to toxicity changes on selected body tissues such as the liver, kidney, and heart. Thus, Fowotade and the team highlighted the potential ability of *A. sativum* extract to induce morphological changes in the liver, kidney, and heart of humans upon the consumption of high doses of garlic for medicinal purposes [16].

Further, the clinical significance, therapeutic utility, and successful commercialization of any herbal drug would depend not only on its therapeutic efficacy but also on its lack of toxicity [17]. Therefore, toxicity studies are equally important to ensure the safety of traditional herbal drugs within a scientific platform to be used as long-term therapeutics and exemplify the possible toxic effects in detail. Toxic effects of plants contain an array of events varying from negligible to severe. Comprehensive toxicity studies facilitate to identify the margin of usage of polyherbal formulations where they can be recommended with the maximum therapeutic effect without any adverse effects.

We herein report the acute and subchronic toxic effects of a polyherbal drug in healthy Wistar rats and acute hypoglycemic and antihyperglycemic effects of the drug in normoglycemic and streptozotocin-induced diabetic rats, respectively. This is the first report on the toxicity profile of the aforementioned antidiabetic drug at a preclinical level.

## 2. Methods

### 2.1. Chemicals and Instruments

Streptozotocin (STZ) was purchased from MP Biomedicals, France. Acetone (Pubchem CID: 180), citric acid, and sodium citrate were of analytical grade (Sigma Chemicals Co, USA) and used without any further purification. The biochemical parameters were estimated using UV-Visible spectrophotometer (Gallenkamp PLC, UK). A tissue processor (Shandon, UK) and a light microscope (Olympus CX, Japan) were used for the preparation of H&E stained tissues and histopathology assessments, respectively. A microtiter plate reader (ELx800, Bio-Tek, Instruments INC, Canada.) was used to measure the absorbance at 450 nm for ELISA measurements. An automated hematological analyzer (Sysmax KH21, Japan) was used in reading hematological parameters.

### 2.2. Plant Materials

The fresh leaves of *M. koenigii,* cloves of *A. sativum,* dried fruit rinds of *G. quaesita*, and dried seeds of *P. nigrum* were collected from the Central Province, Sri Lanka, in March 2017. The plant parts were taxonomically authenticated by comparing specimens at the National Herbarium, National Botanical Garden, Peradeniya, Sri Lanka. The voucher specimens (No: 6/01/H/03) were deposited at National Herbarium, Royal Botanical Garden, Peradeniya, Sri Lanka.

### 2.3. Preparation of the Drug

The polyherbal drug was prepared by grinding an equal amount of dried plant materials (100 g each) and extracted into cold water and water : acetone (1 : 1) separately in an electrical shaker at room temperature (150 rpm, 27°C). Hot water extract of the drug was prepared by refluxing 100 g of plant mixture (25 g of each plant) in 500 mL water at 100°C for three hours. Solvents were evaporated using a rotary evaporator at 40°C. Extracts were further dried by freeze drying to obtain a fine powder of each extract of the drug and it was stored at 0°C until further use.

### 2.4. Experimental Animals

Adult male Wistar rats (body weight: 200 ± 20 g, 10–12 weeks) purchased from Medical Research Institute (MRI), Colombo, Sri Lanka, were used in all experiments. The animals were housed in the Animal Research Facility Centre, Faculty of Medicine, University of Ruhuna, Sri Lanka, at approximate 12 h/12 h of light and dark cycle, temperature at 25°C, and at relative humidity of 65 mmHg. Pelleted food and free water were given *ad libitum* [18]. Adult male Wistar rats were allowed to acclimatize for a period of two weeks prior to be used in experiments. The animals were kept in stainless cages and received humane care. Oral feeding was performed using a stainless steel gavage (Straight, 10 Gauge, 5.9 inches 15.2 cm length, 6.4 mm tip). Animals were handled by trained research and technical staff. Ethical clearance for the animal study was approved by the Ethical Review Committee of Faculty of Medicine, University of Ruhuna, Sri Lanka (09.03.2016.3.8).

### 2.5. Experimental Procedure

#### 2.5.1. Protocol 1: Acute Toxicity Study

The hot water and water : acetone extract were selected for the acute and subchronic toxicity study as those extracts showed potent glucose lowering effect than the cold water extract. The acute toxicity assessment was performed according to the Organization of Economic Cooperation and Development (OECD) guideline 420 for testing of chemicals [19]. Healthy male Wistar rats were randomly divided to three main groups. The first group served as the untreated healthy control group of rats (*n* = 6/group) and received distilled water. The rats of the group two and three were further divided into five subgroups for graded doses of hot water and water : acetone extract of the polyherbal drug at 0.25, 0.5, 1.0, 1.5, and 2.0 g/kg (*n* = 6/subgroup). After a single oral administration of the drugs, rats were observed for 24 hours and once daily for a period of 14 days. The rats were weighed and observed for mortality, salivation, diarrhea, asthenia, hypoactivity, hyperactivity, piloerection, hyperventilation, aggressiveness, yellowing or loss of hair and skin fur, drowsiness, convulsions, tremors, and dizziness.

#### 2.5.2. Protocol 2: Subchronic Toxicity Study

Wistar rats were randomly divided into three main groups (*n* = 6/group). The first group served as the untreated healthy control group and received distilled water daily. The Wistar rats in the second group (*n* = 6/group) received the hot water extract of the polyherbal drug at 1.0 g/kg (equivalent human therapeutic dose). The third group of rats was in three subgroups for three doses selected (*n* = 6/subgroup), and they received the water : acetone extract of the polyherbal drug at doses 0.5, 1.0, and 1.5 g/kg. The oral administration of the polyherbal drug was continued for a period of 28 consecutive days. Fasted rats (12 h) were euthanized on the 28^th^ day with an overdose of aesthetic ether. Subsequently, blood was collected by cardiac puncture and serum was separated for the estimation of biochemical parameters. Kidney and liver tissues were excised for the preparation of H&E stained sections.

The cold water extract was not used for the toxicity studies as it has not shown potent glucose lowering effect compared to the hot water and water : acetone extract.

*(1) Estimation of Biochemical and Hematological Parameters in Healthy Rats*. Serum concentrations of alanine aminotransferase (ALT, EC 2.6.1.2) [20], aspartate aminotransferase (AST, EC 2.6.1.1) [21], alkaline phosphatase (ALP, EC 3.1.3.1) [21], total protein [22], urea [23], and creatinine [24] were estimated using spectrophotometric assay kits. Additional biochemical parameters such as fasting serum concentrations of glucose [25], total cholesterol (TC) [26], high-density lipoprotein cholesterol (HDL-C), and triacylglyceride (TG) [27] were also estimated using spectrophotometric assay kits. The serum concentration of low-density lipoprotein cholesterol (LDL-C) and very low-density lipoprotein cholesterol (VLDL-C) was calculated by using the Friedewald equation [28]. Hematological parameters were estimated using an automated hematological analyzer.

*(2) Histopathological Evaluation of the Liver and Kidney Tissues*. Liver and kidney tissues of all euthanized rats were excised and preserved in 10% buffered formalin. Small slices of the organs were cut by a microtome, stained with hematoxylin-eosin, and examined under a light microscope. Photomicrographs of the liver and kidney tissues of the H&E stained sections were recorded.

*(3) Measurement of Relative Organ Weight*. The heart, lung, small intestine, liver, spleen, pancreas, stomach, and kidney were excised for the assessment of the relative weight of organs. The relative organ weight (ROW) of the heart, lung, small intestine, liver, spleen, pancreas, stomach, and kidney of each animal was calculated by the weight of the organ divided by the bodyweight of the animal on the day they were euthanized.

*(4) Measurements of Consumption of Food and Intake of Water*. The consumption of food and intake of water were measured daily from the quantity of food and water supplied and the amount remaining after 24 hours. The body weight of each rat in all groups was measured during the experimental period at weekly intervals and on the day of sacrifice.

#### 2.5.3. Protocol 3: Effect of Polyherbal Drug on Normoglycemic Rats

Fasted (12 hours) healthy male Wistar rats were randomly divided into four groups based on their body weight. Group one (*n* = 6/group) served as a healthy untreated group and received distilled water. Cold water, hot water, and water : acetone extracts of the polyherbal drug were administered orally to healthy male Wistar rats in groups two, three, and four, respectively. Each test group except the healthy control group (group one) was subdivided into three and received different doses of the polyherbal drug at 0.5 g/kg (*n* = 6/subgroup), 1.0 g/kg (*n* = 6/subgroup), and 1.5 g/kg (*n* = 6/subgroup) as a single dose. Oral glucose tolerance test (OGTT) was performed in all groups as mentioned below.

Glucose (3.00 g/kg) was orally administered to all experimental rats 30 minutes after the administration of polyherbal drug, and blood samples were collected from the tail tip at first, second, third, and fourth hours after the administration of glucose solution. The fasting serum glucose concentration was estimated using a spectrophotometric enzyme assay kit [25]. After single dose administration, acute hypoglycemic activity was evaluated in oral glucose tolerance test over a four hour period using area under the oral glucose tolerance curve.

#### 2.5.4. Protocol 4: Effect of Polyherbal Drug on Diabetic Rats

Diabetes was induced in Wistar rats with an intraperitoneal injection of streptozotocin (STZ), at a dose of 70 mg/kg, dissolved in 0.1 M citric acid/sodium citrate buffer, pH 4.5 [6]. After 72 h of the administration, the fasting serum glucose (FSG) concentration of all animals was estimated using commercially available assay kit. The rats with an FSG of >11.1 mmol/L were regarded as diabetic and were used in experiments starting from the following day after confirmation of diabetes mellitus.

Wistar rats were randomly divided into six groups. Group one (*n* = 6/group) served as the healthy untreated group and group two (*n* = 6/group) served as the diabetic untreated control group which received distilled water. Cold water, hot water, and water : acetone extracts of polyherbal drug were administered orally to diabetic rats in groups three, four, and five, respectively. Each group was subdivided into three and received different doses at 0.5 g/kg (*n* = 6/subgroup), 1.0 g/kg (*n* = 6/subgroup), and 1.5 g/kg (*n* = 6/subgroup). Glibenclamide powder (0.5 mg/kg dose) was dissolved in warm distilled water (37°C) and administered to diabetic rats in the sixth group (*n* = 6/group) which served as the positive control. The oral glucose tolerance test (OGTT) was performed in all groups at 30 minutes after single dose administration of polyherbal drug as described previously.

### 2.6. Statistical Analysis

The biochemical parameters in polyherbal drug treated and control group rats were analyzed using the Minitab statistical software. Comparisons among different groups were performed by using the ANOVA followed by Dunnett's multiple comparison tests. All quantitative data were expressed as mean ± SEM. Statistical significance was considered at *p* < 0.05.

## 3. Results

### 3.1. Acute Toxicity

The acute toxicity study was performed for 14 days to evaluate the adverse/toxic effects produced by a single exposure of the polyherbal drug in a graded range of doses. The hot water and water : acetone extracts of the polyherbal drug did not lead to mortality or any adverse and alternations such as salivation, diarrhea, asthenia, hypoactivity, hyperactivity, piloerection, hyperventilation, aggressiveness, yellowing or loss of hair and skin fur, drowsiness, convulsions, tremors, and dizziness throughout the experimental period.

### 3.2. Subchronic Toxicity

The biochemical, hematological, and histopathological assessments were conducted in evaluating the subchronic toxicity effects of the polyherbal drug. There were no significant alterations in ALT, AST, and ALP enzymes for both hot water and water : acetone extract treated rats and healthy control rats (*p* > 0.05), which suggests that the polyherbal drug might not have hepatotoxic effects and may preserve normal hepatocellular functions at the selected doses (Table 1). The hot water and water : acetone extracts did not produce any significant alterations in the serum concentrations of total protein, urea, and creatinine in polyherbal drug treated rats compared to the healthy control rats (*p* < 0.05). These results indicated that regular kidney functions were maintained normally upon the administration of both extracts in healthy Wistar rats.

Abnormalities in the serum concentration of lipid parameters offer descriptive information on the irregularity of lipid metabolism. The concentration of serum lipid parameters in polyherbal drug treated and healthy control rats is listed in Table 1. The oral administration of the hot water extract at the therapeutic dose lowered the total cholesterol (TC), low-density lipoprotein cholesterol (LDL-C), triglycerides (TG), and very low-density lipoprotein cholesterol (VLDL-C) by 10.61%, 3.31%, 4.17%, and 19.4%, respectively, while showing an increment in serum high-density lipoprotein cholesterol (HDL-C) concentration by 7.7 % (*p* < 0.05). The water : acetone extract of the polyherbal drug significantly reduced the TC, LDL-C, TG, and VLDL-C by 22.3%, 20.9%, 9.2%, and 26.8%, respectively, whereas the serum HDL-C concentration was increased by 16.3% at the therapeutic dose. The results emphasize the modest activity against dyslipidemia in healthy rats, and we postulate that the potency of correcting abnormal lipid alterations would be better in an animal model with dyslipidemia rather than in healthy Wistar rats. The polyherbal drug produced a significant reduction in serum glucose concentration in hot water and water : acetone extracts treated rats compared to healthy control rats by 10.4% and 14.5%, respectively (*p* < 0.05).

The results of hematological parameters in polyherbal drug treated rats are shown in Table 2. There was no significant difference between polyherbal drug-treated rats and untreated healthy control rats in selected hematological parameters: hemoglobin, white blood cell counts, and platelet counts. Hence, all of the above hematological results justified the safety of hot water and water : acetone extracts of the polyherbal drug *in vivo*.

**Figure 1:**
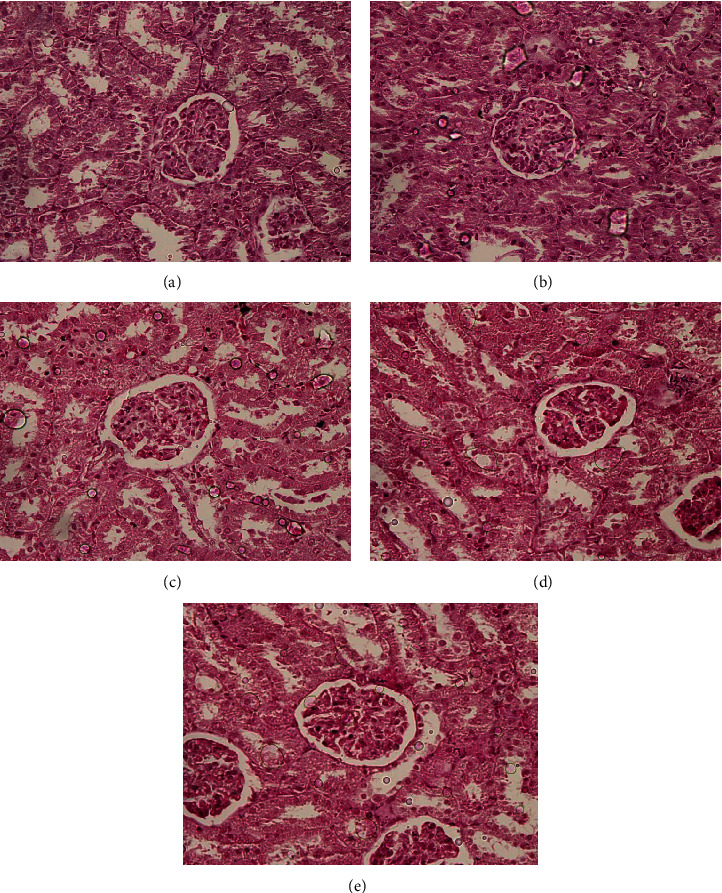
Representative photomicrographs of the kidney in Wistar rats after 28 days of oral administration of the polyherbal drug (×100). (a) Kidney tissue of the healthy control group which received distilled water. (b) Kidney tissues of the rats treated with hot water extract (1.0 g/kg). (c) Kidney tissues of the rats treated with water : acetone extract (0.5 g/kg). (d) Kidney tissues of the rats treated with water : acetone extract (1.0 g/kg). (e) Kidney tissues of the rats treated with water : acetone extract (1.5 g/kg).

**Figure 2:**
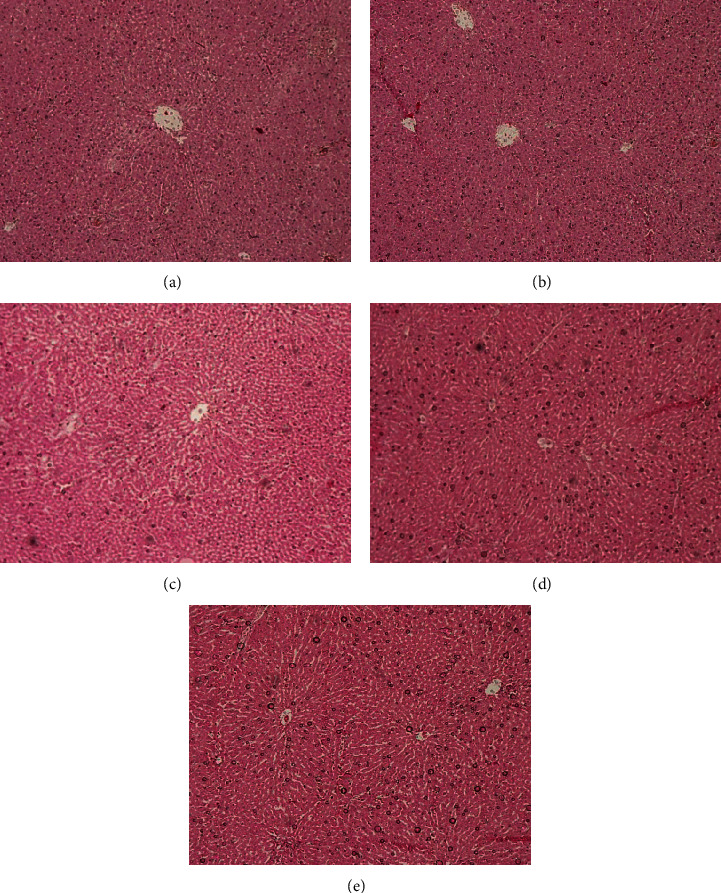
Representative photomicrographs of the liver tissues in Wistar rats after 28 days of oral administration of the polyherbal mixture (×100). (a) Liver tissues of the healthy control group which received distilled water. (b) Liver tissues of the rats treated with hot water extract (1.0 g/kg). (c) Liver tissues of the rats treated with water : acetone extract (0.5 g/kg). (d) Liver tissues of the rats treated with water : acetone extract (1.0 g/kg). (e) Liver tissues of the rats treated with water : acetone extract (1.5 g/kg).

The histopathological assessment is crucial in evaluating toxicity-related changes of herbal drugs and has been considered as the gold standard. The photomicrographs of histopathological changes of the H&E stained kidney and liver sections of the experimental animals are shown in Figures 1 and 2, respectively. The oral administration of the hot water and water : acetone extracts of the polyherbal drug did not produce any harmful or abnormal conditions in the kidney and liver tissues. H&E stains of kidney tissues showed normal structural features suggesting the preservation of renal integrity in the treated rats. The glomeruli and renal tubules exhibit normal architecture indicating the absence of renal toxicity in plant extract treated rats. There were no significant alterations in arrangement of hepatocytes and no inflammatory or necrotic changes in polyherbal drug treated rats compared to the healthy control rats. Moreover, no granuloma or malignancy was evident in the liver tissues after an oral administration of the polyherbal drug for subchronic period. However, the genotoxicity and carcinogenicity assessments are also recommended in advance to evaluate the effect of the aforementioned polyherbal drug on chromosomes, genes, and cancer development prior to the conductance of clinical studies.

The measurement of relative organ weight also serves as a sensitive tool in toxicity studies. Changes in the relative organ weight are considered as signs of adverse/toxic effects to vital organs in subchronic and chronic toxicological experiments.  Table 3 illustrates the effect of the both extracts of the polyherbal drug on relative organ weights of excised organs in experimental rats. There is no significant difference between the relative organ weight of the excised heart, lungs, spleen, pancreas, liver, kidney, stomach, and small intestine in plant extract treated rats and healthy control rats after 28 days of experimental period (*p* > 0.05) (Table 3). The heart, liver, kidneys, spleen, and lungs are the primary organs which basically indicate the effects of toxic substances in metabolic reactions. The absence of significant changes in the relative organ weight of primary organs indicates that the polyherbal drug did not lead to gross alterations in metabolic reactions on prolonged usage.

The effects of polyherbal drug on body weight gain, consumption of food, and intake of water in experimental rats at weekly intervals are shown in Tables 4–6, respectively. Although there was no significant difference in body weight gain in polyherbal drug treated rats compared to the healthy control rats during the first two weeks (*p* > 0.05), a significant reduction in body weight gain was demonstrated in polyherbal drug treated rats from the second week onwards. However, there is no significant difference (*p* < 0.05) in consumption of food and intake of water in plant extract treated rats and healthy control rats which suggests that polyherbal drug might not interfere with the overall metabolic processes in healthy rats.

### 3.3. Effect of Polyherbal Drug on Normoglycemic Rats and Diabetic Rats

The results of the area under the glucose tolerance curve values upon the administration of cold water, hot water, and water: acetone extracts of the polyherbal drug to healthy and diabetic rats are shown in Tables 7 and 8, respectively. Polyherbal drug treated healthy rats did not reach a severe reduction in area under the curve values compared to the untreated group. However, the hypoglycemic effect increases with dose in both groups of hot water and water: acetone extracts treated rats as 1.5 g/kg dose resulted in maximum glucose lowering effect. Between two extracts, water: acetone extract showed maximum hypoglycemic effect than hot water extract in healthy rats. The oral administration of cold water, hot water, and water: acetone extracts produced an overall reduction in the total area under the curve, after a four hour period of oral glucose tolerance test compared to the diabetic rats (*p* < 0.05). Low total area under the curve reflects high efficacy or improvement on glucose tolerance of the particular drug. The hot water and water: acetone extracts of the polyherbal drug showed a statistically significant improvement on total area under the glucose tolerance curve (TAUC) in streptozotocin-induced diabetic rats at the doses of 1.0 and 1.5 g/kg (*p* < 0.05). The highest dose (1.5 g/kg) of each group resulted in the maximum antihyperglycemic effect in diabetic rats while water: acetone extract of polyherbal mixture showed higher antihyperglycemic effect than the hot water extract. Further water: acetone extract of polyherbal drug at 1.5 g/kg dose showed potent blood glucose lowering effect which is closer to the antidiabetic effect of positive control, glibenclamide.

## 4. Discussion

Diabetes and its associated complications have become devastating health problems at present and millions of people are affected with this dilemma worldwide. Although synthetic drugs are capable of treating diabetes mellitus for a certain extent, popularity of these drugs is being overshadowing by unwanted side effects [29]. Therefore, alternative therapeutic approaches to manage diabetes and its complications have gained attention among the general public particularly in South Asian countries. Since 4000 years, Sri Lankan traditional medicine has been extensively practiced in each part of the country [30]. Various polyherbal drugs that have been prescribed by traditional practitioners provide promising solutions for the management of diabetes and dyslipidemia/hypercholesterolemia [31]. However, safety profile of these commercially viable herbal drugs should be investigated in a scientifically rigorous fashion with properly conducted *in vivo* studies. Indeed, absence of toxicity provides confidence for recommending polyherbal remedies in clinical practice if these are being able to prove the efficacy.

Toxic or adverse effects are influenced by the rate at which the pharmacological agents enter the body and how they interact with various body tissues including vital organs such as the liver and kidney. Studies in whole animals are critical to ensure the proper use of the beneficial medicines as laboratory tools simply cannot duplicate complicated body phenomena [32]. In the present study, we investigated the acute hypoglycemic and antihyperglycemic activities and detailed toxicity assessment of a polyherbal drug composed of leaves of *M. koenigii*, cloves of *A. sativum*, dried fruit rinds of *G. quaesita*, and seeds of *P. nigrum* in Wistar rats which have been used in the treatment of diabetes mellitus and dyslipidemia.

Toxicity assessment of hot water and water: acetone extracts of the polyherbal drug was conducted in healthy Wistar rats. Male Wistar rats were used as sensitivity of males is greater than that of females in most of the routine renal and liver toxicity parameter measurements [33]. In the toxicity assessment, the hot water extract and the water : acetone extract of the polyherbal drug were used to simulate the clinical use of the drug in present therapeutic applications in traditional medicine and to evaluate potential toxic effects in the extracts with highest antihyperglycemic effects, respectively. Based on the results of the toxicity evaluation, the lethal dose (LD_50_) of the hot water and water:acetone extracts was assumed to be greater than 2.0 g/kg, suggesting that the polyherbal drug could be generally considered as nontoxic at the human equivalent therapeutic dose. In the subchronic toxicity study, the therapeutic dose of 1.0 g/kg was used for the administration of the hot water extract, while three doses were selected as 0.5, 1.0, and 1.5 g/kg for the most active water: acetone extract, with an aim of evaluating the dose-dependent toxic effects, if any. Indeed, therapeutic dose of drug administration involves measuring drug concentrations to facilitate the clinical interpretation of the results [34].

The assessment of biochemical parameters reflects possible toxic effects of herbal drugs towards hepatic and renal functions. The assessment of liver and kidney functions is crucial as vital organs in the body have multifarious mechanisms to eliminate toxic substances through the liver and kidney in order to reduce their toxic effects. Even though liver and kidney are remarkable in eliminating toxins, their functions can be damaged during the toxin purgation process [35]. In this regard, several biochemical parameters are critical in the evaluation of hepatic dysfunction and liver cell damage. ALT, AST, and ALP are routine liver enzymes which can be used to screen the alterations of the liver functions [36]. Quantitative estimation of kidney function parameters in experimental rats directly affects the drug dosing and enables clinical trials of novel therapeutic agents [37]. Urea and creatinine are first-line screening tests of renal functions [38]. Evaluation of serum total protein also gives an estimation of the nutritional status and diagnostic measurement for alternations in kidney functions [39]. The dysfunction of kidney leads to inefficient excretion of both urea and creatinine causing their accumulation in the blood. Based on our results, it is revealed that the water and water:acetone extracts of the polyherbal drug did not cause liver toxicity and renal toxicity in healthy rats, and we postulate that the prescription of the drug might be safe for patients with diabetes and dyslipidemia; however, clinical data are needed to confirm the results.

Thus, beneficial changes in the serum concentration of glucose, TC, HDL-C, LDL-C, TG, and VLDL-C reported in this study might suggest the hypoglycemic and hypolipidemic potential of the combined plant mixture. Even though fasting serum glucose concentration and lipid parameter values are not involving directly in elucidating toxic effects, these are important in eliciting the antihyperlipidemic efficacy for a certain extent. However, detailed mechanistic approach is warranted to explore detailed antidiabetic cellular mechanisms.

Evaluation of hematological parameters is used to determine the extent of the deleterious effect of the tested extract on the blood-related functions in experimental healthy rats. The results of the present study revealed that there were no significant effects on erythropoiesis, morphology, or osmotic fragility of RBC [40]. Similarly, the absence of significant changes in white cell counts between polyherbal drug treated rats and healthy control rats reflected the intact condition of the immune system and lack of damages to the tissues [41]. Moreover, insignificant alterations in platelets count in plant extract treated rats suggested the absence of the substantial effects on fibrin fibers and damages to the blood vessels [42].

The histology of the liver and kidney tissues are considered as vital tools for illustrating liver and kidney damages by toxic substances [43]. The toxicity-related parameters as interstitial edema, epithelial changes, tubular degeneration, capillary congestion, and leukocyte infiltration were examined [44, 45]. The detailed subchronic toxicity evaluation confirmed the fact that the polyherbal drug did not cause adverse and/or toxic effects through biochemical, hematological, and histological assessment; therefore, the drug seems to be promising in clinical use.

The body weight changes normally revealed a proper nutritional status of experimental rats. Body weight is an important anthropometric parameter in the assessment of overall nutrition [46]. Previous studies revealed that individual plant extract which is in a combined plant mixture can exert weight loss by several mechanisms. According to the report by Kim et al., cloves of *A. sativum* can decrease the cell size of white adipose tissue in order to suppress the body weight gain [47]. Allicin which is a main compound of *A. sativum* has improved the metabolism in high-fat diet-induced obese mice by regulating the composition of the intestinal microbiota [48]. Antiobesity and lipid lowering effects of leaves of *M. koenigii* and its isolated compound, mahanimbine, have been reported with significant reduction of body weight gain [49]. Further, garcinol, a prominent antioxidant compound present in *G. quaesita*, shows promising effects on reduction of body weight [50]. Therefore, it can be assumed that hot water and water: acetone extracts of the polyherbal mixture led to a reduction in body weight due to combined effect of many active compounds such as garcinol, mahanimbine, allicin, and piperine in plant extract treated rats than in untreated healthy control rats.

Recent studies have shown that carbazole alkaloids are the major phytochemical class present in *M. koenigii* that are responsible for hypoglycemic activity. Among them, mahanimbine, koenidine, and koenimbine have potent hypoglycemic activities which enhance insulin effect in diabetic individuals through increasing the peripheral glucose uptake or secretion by islets of Langerhans of pancreatic beta cells [51, 52]. Moreover, secondary metabolites present in *A. sativum* such as S-allylcysteine have allicin which has the potential to reduce the serum glucose concentration comparable to glibenclamide through insulin secretion mechanism [53, 54]. Garcinol which is a potent antidiabetic compound is isolated from *G. quaesita*, and it could assist in generation of insulin-producing *β* cells [55–57]. Piperine which is the most abundant alkaloid in *P. nigrum* has potent antidiabetic activity and has the ability to inhibit the biotransformation process of compounds in the liver and gastrointestinal track during metabolic process [58, 59]. Hence, piperine could preserve the important antidiabetic chemical moieties of the ingredients of this polyherbal drug by acting as a bioenhancer. Therefore, the aforementioned polyherbal remedy could provide antihyperglycemic activity through improvement in insulin secretion and regeneration of pancreatic *β* cells as possible antidiabetic mechanisms based on published reports.

## 5. Conclusions

Acute toxicity study showed that the polyherbal drug did not cause any change in animals throughout the experimental period of 14 days. The lethal dose (LD_50_) of the polyherbal drug was assumed to be > 2.0 g/kg as no mortality was observed in the acute toxicity study. The absence of significant alterations in biochemical parameters, hematological parameters, and relative organ weights and histopathology of H&E stained sections of the liver and kidney tissues of hot water and water: acetone extracts of polyherbal drug confirmed its *in vivo* safety for the first time. In addition, the results of the glucose tolerance test revealed that the polyherbal drug composed of leaves of *M. koenigii* (L.), cloves of *A. sativum* (L), dried fruits of *G. quaesita* Pierre, and dried seeds of *P. nigrum* (L) possesses potent antihyperglycemic activity in streptozotocin-induced diabetic rats and did not cause severe hypoglycemia in healthy Wistar rats.

## Figures and Tables

**Table 1:** Effect of the polyherbal drug on selected biochemical parameters in healthy rats after 28 days.

Biochemical parameters	Treatments				
	Healthy control	Hot water extract (1.0 g/kg)	Water : acetone extract (0.5 g/kg)	Water : acetone extract (1.0 g/kg)	Water : acetone extract (1.5 g/kg)
ALT (U/L)	59.66 ± 1.61	61.88 ± 1.17	61.46 ± 1.34	60.02 ± 1.88	62.03 ± 1.62^*∗*^
AST (U/L)	42.43 ± 1.52	44.05 ± 1.20	42.38 ± 1.05	42.69 ± 0.92	42.87 ± 1.40
ALP (U/L)	149.54 ± 1.26	147.78 ± 1.30	147.42 ± 0.94	146.61 ± 1.59	147.29 ± 1.03
Creatinine (mmol/L)	22.16 ± 0.08	23.30 ± 0.07^*∗*^	22.68 ± 0.06	21.96 ± 0.08	22.86 ± 0.08
Urea (mmol/L)	18.98 ± 1.60	19.51 ± 1.17	18.24 ± 1.41	18.35 ± 1.23	19.01 ± 1.37
Total protein (mmol/L)	7.82 ± 0.17	7.86 ± 0.28	7.54 ± 0.24	8.16 ± 0.30	7.95 ± 0.27
TC (mg/dL)	5.18 ± 0.01	4.63 ± 0.01^*∗*^	4.47 ± 0.04^*∗*^	4.02 ± 0.01^*∗*^	3.80 ± 0.02^*∗*^
HDL-C (mg/dL)	3.11 ± 0.03	3.35 ± 0.02^*∗*^	3.24 ± 0.01^*∗*^	3.62 ± 0.01^*∗*^	3.81 ± 0.02^*∗*^
LDL-C (mg/dL)	1.81 ± 0.01	1.75 ± 0.01^*∗*^	1.62 ± 0.02^*∗*^	1.43 ± 0.07^*∗*^	1.24 ± 0.03^*∗*^
TG (mg/dL)	3.35 ± 0.02	3.21 ± 0.06^*∗*^	3.19 ± 0.02^*∗*^	3.04 ± 0.01^*∗*^	2.90 ± 0.01^*∗*^
VLDL-C (mg/dL)	0.67 ± 0.01	0.54 ± 0.02^*∗*^	0.41 ± 0.08^*∗*^	0.49 ± 0.02^*∗*^	0.22 ± 0.01^*∗*^
Fasting glucose (mmol/L)	5.17 ± 0.62	4.63 ± 0.31^*∗*^	4.71 ± 1.02	4.42 ± 0.92^*∗*^	4.32 ± 0.47^*∗*^

Each data point is expressed as mean ± SEM (*n* = 6/group). ALT: alanine aminotransferase, AST: aspartate aminotransferase, ALP: alkaline phosphatase, TC: serum concentration of total cholesterol, HDL-C: serum concentration of high-density lipoprotein, TG: triacylglyceride, LDL-C: the serum concentration of low-density lipoprotein cholesterol, and VLDL-C: the serum concentration of very low-density lipoprotein cholesterol. Results are significant compared to the control group at ^*∗*^*p* < 0.05.

**Table 2:** Effect of plant extracts on hematological parameters in healthy rats after 28 days.

Hematological parameter	Treatments				
	Healthy control rats	Hot water extract (1.0 g/kg)	Water : acetone extract (0.5 g/kg)	Water : acetone extract (1.0 g/kg)	Water : acetone extract (1.5 g/kg)
Basophils (%)	31.13 ± 4.89	30.00 ± 4.53	30.73 ± 3.92	33.23 ± 4.02	32.48 ± 5.01
Eosinophils (%)	2.27 ± 0.27	1.52 ± 0.32	1.63 ± 0.18	1.65 ± 0.41	1.79 ± 0.47
Hemoglobin (g/dL)	14.56 ± 0.23	15.60 ± 0.28	15.70 ± 0.16	15.71 ± 0.39	15.92 ± 0.58
HCT (%)	48.65 ± 1.59	53.72 ± 2.35	52.67 ± 2.93	53.63 ± 1.82	53.32 ± 2.03
Lymphocytes (%)	63.18 ± 4.31	75.72 ± 4.29	71.12 ± 3.60	72.25 ± 4.58	70.89 ± 2.40
MCH (pg)	17.70 ± 0.29	17.40 ± 0.45	17.54 ± 0.34	17.63 ± 0.51	17.35 ± 0.46
MCHC g/dL	30.23 ± 0.43	29.78 ± 0.33	30.05 ± 0.16	29.26 ± 0.82	29.88 ± 0.64
MCV (fl)	58.68 ± 0.19	58.72 ± 0.11	58.82 ± 0.23	60.31 ± 0.34	59.18 ± 0.22
Neutrophil (%)	6.14 ± 2.1	5.53 ± 0.39	5.80 ± 0.20	4.14 ± 0.35	5.52 ± 0.41
Platelet count (/cu.mm)	505200.00 ± 3346	526600.00 ± 4571	510450 ± 3683	430840.00 ± 4123	4859200 ± 3620
RDW (%)	10.33 ± 0.17	10.58 ± 0.11	10.45 ± 0.24	10.54 ± 0.37	10.51 ± 0.26
WBC (/cu.mm)	4980.00 ± 376	6358.00 ± 420	4872.00 ± 297	2859.00 ± 411	5329.00 ± 380
Monocytes (%)	0.71 ± 0.16	0.65 ± 0.10	0.73 ± 0.29	0.75 ± 0.31	0.69 ± 0.40

Each data point is expressed as mean ± SEM (*n* = 6/group. Results are significant compared to the control group at ^*∗*^*p* < 0.05.

**Table 3:** Effect of the polyherbal drug on relative organ weight of organs in healthy rats after 28 days.

Organ	Relative organ weight				
	Healthy control	Hot water extract (1.0 g/kg)	Water : acetone extract (0.5 g/kg)	Water : acetone extract (1.0 g/kg)	Water : acetone extract (1.5 g/kg)
Heart	0.29 ± 0.01	0.29 ± 0.01	0.28 ± 0.01	0.28 ± 0.01	0.28 ± 0.01
Lungs	0.41 ± 0.01	0.45 ± 0.01	0.42 ± 0.01	0.43 ± 0.01	0.43 ± 0.01
Spleen	0.20 ± 0.01	0.20 ± 0.03	0.20 ± 0.02	0.20 ± 0.01	0.20 ± 0.03
Pancreas	0.32 ± 0.01	0.32 ± 0.01	0.32 ± 0.01	0.33 ± 0.01	0.32 ± 0.01
Liver	2.55 ± 0.07	2.45 ± 0.13	2.53 ± 0.08	2.47 ± 0.05	2.49 ± 0.06
Kidney	0.56 ± 0.02	0.57 ± 0.02	0.56 ± 0.01	0.59 ± 0.01	0.56 ± 0.01
Stomach	0.94 ± 0.06	0.74 ± 0.04	0.79 ± 0.06	0.93 ± 0.01	0.84 ± 0.08
Small intestine	1.54 ± 0.06	1.60 ± 0.09	1.62 ± 0.05	1.62 ± 0.06	1.55 ± 0.06

Each data point is expressed as mean ± SEM (*n* = 6/group). Results are significant compared to the control group at ^*∗*^*p* < 0.05.

**Table 4:** Changes in the body weight of Wistar rats (subacute or subchronic toxicity study).

Treatments	Body weight (g)			
	1^st^ week	2^nd^ week	3^rd^ week	4^th^ week
Healthy control rats	268.15 ± 3.72	277.12 ± 2.53	285.50 ± 3.42	292.18 ± 4.01
Hot water extract treated rats (1.0 g/kg)	266.60 ± 2.03	268.32 ± 3.05	274.64 ± 2.83	280.52 ± 2.12
Water : acetone extract treated rats (0.5 g/kg)	264.64 ± 1.20	268.42 ± 2.94	270.36 ± 2.58	278.20 ± 3.01
Water : acetone extract treated rats (0.5 g/kg)	268.70 ± 2.54	274.16 ± 3.47	276.78 ± 3.79	280.38 ± 2.69
Water : acetone extract treated rats (1.5 g/kg)	266.50 ± 2.48	268.14 ± 4.06	269.24 ± 1.94	270.62 ± 1.73

Each data point is expressed as mean ± SEM (*n* = 6/group). Results are significant compared to the control group at ^*∗*^*p* < 0.05.

**Table 5:** Effect of the polyherbal drug on consumption of food in healthy rats at weekly intervals.

Treatment	Average consumption of food (g/day)			
	1^st^ weak	2^nd^ weak	3^rd^ weak	4^th^ weak
Healthy control rats	22.00 ± 0.59	22.33 ± 0.81	20.50 ± 1.04	20.16 ± 0.93
Hot water extract (1.0 g/kg)	22.50 ± 1.24	22.00 ± 1.52	20.83 ± 0.62	20.33 ± 0.81
Water : acetone extract (0.5 g/kg)	21.66 ± 0.48	22.66 ± 1.02	20.00 ± 1.71	20.66 ± 1.09
Water : acetone extract (0.5 g/kg)	21.33 ± 0.71	20.50 ± 0.34	19.66 ± 0.61	19.91 ± 0.92
Water : acetone extract (1.5 g/kg)	20.83 ± 0.90	20.33 ± 1.69	19.75 ± 1.24	19.58 ± 0.53

Each data point is expressed as mean ± SEM (*n* = 6/group). Results are significant compared to the control group at ^*∗*^*p* < 0.05.

**Table 6:** Effect of the polyherbal drug on intake of water in healthy rats at weekly intervals.

Treatment	Average consumption of water (mL/day)			
	1^st^ weak	2^nd^ weak	3^rd^ weak	4^th^ weak
Healthy control rats	66.66 ± 0.86	62.50 ± 0.92	61.66 ± 1.09	63.33 ± 0.72
Hot water extract (1.0 g/kg)	64.16 ± 0.75	64.16 ± 0.87	63.47 ± 1.12	62.84 ± 1.36
Water : acetone extract (0.5 g/kg)	63.58 ± 1.24	64.90 ± 1.53	62.28 ± 1.03	61.15 ± 1.26
Water : acetone extract (0.5 g/kg)	64.10 ± 1.49	62.73 ± 1.81	63.61 ± 1.42	62.44 ± 1.69
Water : acetone extract (1.5 g/kg)	64.25 ± 0.83	61.47 ± 0.46	62.26 ± 0.80	61.59 ± 0.85

Each data point is expressed as mean ± SEM (*n* = 6/group). Results are significant compared to the control group at ^*∗*^*p* < 0.05.

**Table 7:** Effect of area under the oral glucose tolerance curve values of the polyherbal drug in healthy rats.

Group	Area under the oral glucose tolerance curve values (mmol/L·h)					
	½ h	1 h	2 h	3 h	4 h	Total
Healthy control rats	3.40 ± 0.07	4.38 ± 0.09	7.32 ± 0.16	5.85 ± 0.06	4.57 ± 0.07	25.52 ± 0.45
Cold water extract (0.5 g/kg)	3.56 ± 0.10^*∗*^	4.24 ± 0.12^*∗*^	6.84 ± 0.19^*∗*^	5.77 ± 0.14^*∗*^	5.19 ± 0.07^*∗*^	25.60 ± 0.62^*∗*^
Cold water extract (1.0 g/kg)	3.48 ± 0.07^*∗*^	4.10 ± 0.08^*∗*^	6.32 ± 0.16^*∗*^	5.24 ± 0.05^*∗*^	4.75 ± 0.15^*∗*^	23.89 ± 0.51^*∗*^
Cold water extract (1.5 g/kg)	3.46 ± 0.05^*∗*^	4.07 ± 0.11^*∗*^	6.29 ± 0.20^*∗*^	5.13 ± 0.12^*∗*^	3.94 ± 0.08^*∗*^	22.89 ± 0.56^*∗*^
Hot water extract (0.5 g/kg)	3.40 ± 0.06	4.00 ± 0.08	6.16 ± 0.15^*∗*^	5.21 ± 0.07^*∗*^	4.10 ± 0.13^*∗*^	22.87 ± 0.49^*∗*^
Hot water extract (1.0 g/kg)	3.38 ± 0.08	4.08 ± 0.11	6.41 ± 0.07^*∗*^	4.67 ± 0.13^*∗*^	3.55 ± 0.14^*∗*^	22.09 ± 0.53^*∗*^
Hot water extract (1.5 g/kg)	3.22 ± 0.06	3.95 ± 0.07	5.96 ± 0.20^*∗*^	4.39 ± 0.10^*∗*^	3.64 ± 0.09^*∗*^	21.16 ± 0.52^*∗*^
Acetone : water extract (0.5 g/kg)	3.36 ± 0.09	4.04 ± 0.09	6.27 ± 0.04^*∗*^	4.75 ± 0.14^*∗*^	3.85 ± 0.19^*∗*^	22.27 ± 0.46^*∗*^
Acetone : water extract (1.0 g/kg)	3.38 ± 0.008	4.08 ± 0.11	6.35 ± 0.05^*∗*^	4.62 ± 0.14^*∗*^	3.49 ± 0.11^*∗*^	21.92 ± 0.41^*∗*^
Acetone : water extract (1.5 g/kg)	3.36 ± 0.09	3.93 ± 0.07^a^	5.97 ± 0.04^*∗*^	4.54 ± 0.14^*∗*^	3.46 ± 0.11^*∗*^	21.26 ± 0.38^*∗*^

The values are expressed as mean ± SEM (*n* = 6/group). Data were analyzed by ANOVA followed by Dunnett's test (*p* < 0.05). Results are significant compared to the control group at ^*∗*^*p* < 0.05.

**Table 8:** Effect of area under the oral glucose tolerance curve values of the polyherbal drug in streptozotocin-induced diabetic rats.

Group	Area under the oral glucose tolerance curve values (mmol/L·h)					
	½ h	1 h	2 h	3 h	4 h	Total
Healthy control rats	3.44 ± 0.09	3.93 ± 0.17	6.21 ± 0.24	5.23 ± 0.09	4.74 ± 0.04	23.55 ± 0.63
Diabetic control rats	7.42 ± 0.17	8.26 ± 0.23	15.40 ± 0.41	14.17 ± 0.34	13.05 ± 0.39	58.30 ± 1.54
Diabetic rats + cold water extract (0.5 g/kg)	6.86 ± 0.24^*∗*^	7.67 ± 0.08^*∗*^	14.02 ± 0.13^*∗*^	12.06 ± 0.26^*∗*^	11.13 ± 0.46^*∗*^	51.74 ± 1.17^*∗*^
Diabetic rats + cold water extract (1.0 g/kg)	7.17 ± 0.11^*∗*^	7.86 ± 0.12^*∗*^	13.51 ± 0.21^*∗*^	11.45 ± 0.35^*∗*^	9.60 ± 0.25^*∗*^	49.59 ± 1.04^*∗*^
Diabetic rats + cold water extract (1.5 g/kg)	6.78 ± 0.25^*∗*^	7.00 ± 0.27^*∗*^	12.40 ± 0.46^*∗*^	10.38 ± 0.27^*∗*^	4.25 ± 0.36^*∗*^	40.81 ± 1.61^*∗*^
Diabetic rats + hot water extract (0.5 g/kg)	6.93 ± 0.20	7.50 ± 0.25	13.63 ± 0.39	11.92 ± 0.18	10.60 ± 0.19	50.58 ± 1.21
Diabetic rats + hot water extract (1.0 g/kg)	6.75 ± 0.07	7.11 ± 0.06	11.92 ± 0.19^*∗*^	10.73 ± 0.37^*∗*^	9.96 ± 0.49^*∗*^	46.47 ± 1.18^*∗*^
Diabetic rats + hot water extract (1.5 g/kg)	7.04 ± 0.21	7.09 ± 0.16	11.43 ± 0.30^*∗*^	9.63 ± 0.41^*∗*^	8.34 ± 0.19^*∗*^	43.53 ± 1.27^*∗*^
Diabetic rats + acetone : water extract (0.5 g/kg)	7.23 ± 0.05	7.52 ± 0.14	12.08 ± 0.90^*∗*^	10.86 ± 0.82^*∗*^	10.70 ± 0.32^*∗*^	48.39 ± 2.23^*∗*^
Diabetic rats + acetone : water extract (1.0 g/kg)	7.30 ± 0.18	6.88 ± 0.08^*∗*^	10.72 ± 0.28^*∗*^	9.91 ± 0.30^*∗*^	9.12 ± 0.36^*∗*^	43.94 ± 1.20^*∗*^
Diabetic rats + acetone : water extract (1.5 g/kg)	7.36 ± 0.10	7.02 ± 0.15	10.36 ± 0.21^*∗*^	9.01 ± 0.17^*∗*^	8.24 ± 0.20^*∗*^	41.99 ± 0.83^*∗*^
Diabetic rats + glibenclamide (0.5 mg/kg)	7.19 ± 0.19	6.55 ± 0.20^*∗*^	9.22 ± 0.46^*∗*^	7.48 ± 0.30^*∗*^	6.00 ± 0.36^*∗*^	36.44 ± 1.51^*∗*^

The values are expressed as mean ± SEM (*n* = 6/group). Data were analyzed by ANOVA followed by Dunnett's test (*p* < 0.05). Results are significant compared to the control group at ^*∗*^*p* < 0.05.

## Data Availability

The data used to support the findings of this study are included within the article, and the raw data are also available upon request.
